# Reducing Maternal Mortality Rates in Alabama Through Patient Education: A Scoping Review

**DOI:** 10.7759/cureus.43172

**Published:** 2023-08-08

**Authors:** Rebecca Telese, Andrew D Vogel, Mohammed T Azam, Patrick G Dickinson, Alyssa Zakala, Juanita T Heersink

**Affiliations:** 1 Research, Alabama College of Osteopathic Medicine, Dothan, USA; 2 Internal Medicine, Southeast Health Medical Center, Dothan, USA

**Keywords:** united states of america, alabama, health education & awareness, prevention in primary care, patient education, maternal mortality

## Abstract

Maternal mortality continues to rise in the United States and disproportionately affects those in Alabama. Lack of patient education on warning signs is a preventable cause of maternal mortality. This article aims to systematically quantify existing research investigating the effect of patient education on maternal outcomes. The inclusion criteria required an article to be (a) original research, (b) conducted within the United States, (c) in English, and (d) published between January 2012 and September 2022. PubMed® and Embase® databases were searched using key words and filters. Rayyan®, a systematic review research tool, was utilized to assess articles in a blinded two-person review process. A blinded third researcher resolved conflicts. A total of 3,139 articles were compiled; 3,115 articles did not meet inclusion criteria, and 24 articles were retrieved after an abstract review. Ultimately, 11 articles were included after a full-text review. None of these articles were specific to Alabama. However, they did contain evidence for patient education improving maternal mortality. More research is required in Alabama to demonstrate the effect of educating patients on maternal mortality. These articles contain evidence for education as a tool to improve maternal outcomes.

## Introduction and background

Despite the vast improvements in healthcare, maternal mortality continues to rise in the United States and is disproportionately affecting women in Alabama. In 2020, the United States had a maternal mortality rate of 23.8 deaths per 100,000 live births [1]. That same year, Alabama had a maternal mortality rate of 36.4 deaths per 100,000 live births [2]. There needs to be action taken to address the high rates of women dying due to pregnancy-associated complications. Educating patients on warning signs can be a cost-efficient way of reducing maternal deaths. In 2019, the cost of maternal mortality in the United States was estimated to be $32.3 billion [3]. Reducing the financial burden of maternal mortality would allow funds to be saved and allocated to other health care initiatives.

Though there can be numerous factors associated with these statistics, such as socioeconomic status or physical distance from health care, one aspect is a lack of knowledge among patients about specific warning signs and symptoms associated with serious illness. The Centers for Disease Control and Prevention (CDC) defines maternal mortality as a death while pregnant or up to one year after pregnancy, from anything due to or exacerbated by pregnancy [4]. This definition includes a wide array of potential causes of death that each present differently. While patients and their families may be familiar with certain common symptoms of potentially life-threatening illnesses, it is likely they are unaware of the full range of possible warning signs.

Studies have shown an association between health literacy and adult literacy [5]. Health literacy is a patient's ability to receive and understand information related to their health in order to make informed decisions. Adult literacy is defined as the reading comprehension and writing ability of an adult. In a state such as Alabama, with a low average adult literacy rate, it is critical to take the time to verify that patients comprehend the information being provided to them [6]. Health care providers cannot assume that their patients have knowledge of potential health complications nor understand what steps should be taken if they experience such symptoms.

A report from 2018 compiling data from nine Maternal Mortality Review Committees (MMRCs) cited that 63.2% of maternal deaths were preventable [7]. Hemorrhage, cardiovascular/coronary conditions, cardiomyopathy, or infection contributed to approximately 50% of pregnancy-related deaths (PRDs) [8]. In addition, maternal death affects black women three to four times more than white women in the United States. All these PRDs are associated with warning signs that may be appreciated by patients in their own homes. The MMRCs additionally determined that one of the most common causes contributing to these deaths was inadequate education on warning signs and knowing when to obtain treatment. Something as simple as taking the time to provide information to patients and their families can save countless lives. However, it does not appear that all health care providers are ensuring that such education is accessible to their patients.

This article was previously presented as a poster at Region 4 Physicians of the Future Summit Research Symposium on January 28, 2023, at the Society for Hospital Medicine Research Poster Week on November 29, 2022, and at Alabama College of Osteopathic Medicine Poster Day on December 2, 2022.

## Review

Methods

The inclusion criteria required an article to be (a) original research, (b) conducted within the United States, (c) in English, and (d) published between January 2012 and September 2022. PubMed® and Embase® were selected as databases to search for articles.

Medical Subject Headings (MeSH) terms were developed to be used in the PubMed search and separate specific terms were selected for Embase (Table 1).

**Table 1:** Search terms for each database

Database	Search Terms
PubMed	("Maternal Death"[MeSH Terms] OR "Maternal Mortality"[MeSH Terms] OR "Maternal Health"[MeSH Terms]) AND ("Maternal Health Services"[MeSH Terms] OR "Prenatal Education"[MeSH Terms] OR "Health Education"[MeSH Terms] OR "Patient Education as Topic"[MeSH Terms] OR "Preventive Health Services"[MeSH Terms] OR "Teaching Materials"[MeSH Terms] OR "Primary Prevention"[MeSH Terms]) AND "United States"[MeSH Terms]
Embase	('maternal mortality'/exp OR 'maternal mortality' OR 'maternal death') AND ('maternal care' OR 'maternal health service' OR 'patient education' OR 'patient education material' OR 'health education' OR 'teaching' OR 'education' OR 'prevention') AND 'united states'

The PubMed and Embase searches yielded 3,139 articles in total. Rayyan®, a systematic review research tool, was then utilized to assess all the articles in a blinded two-person review process. Two researchers were assigned for a preliminary abstract review of the PubMed articles and another two for Embase. Each researcher read the abstract and determined whether to include or exclude each article. For conflicts, a blinded third researcher would decide on article inclusion or exclusion. A total of 3,115 articles did not meet the inclusion criteria following the preliminary review, meaning that 24 articles were sought for retrieval.

**Figure 1:**
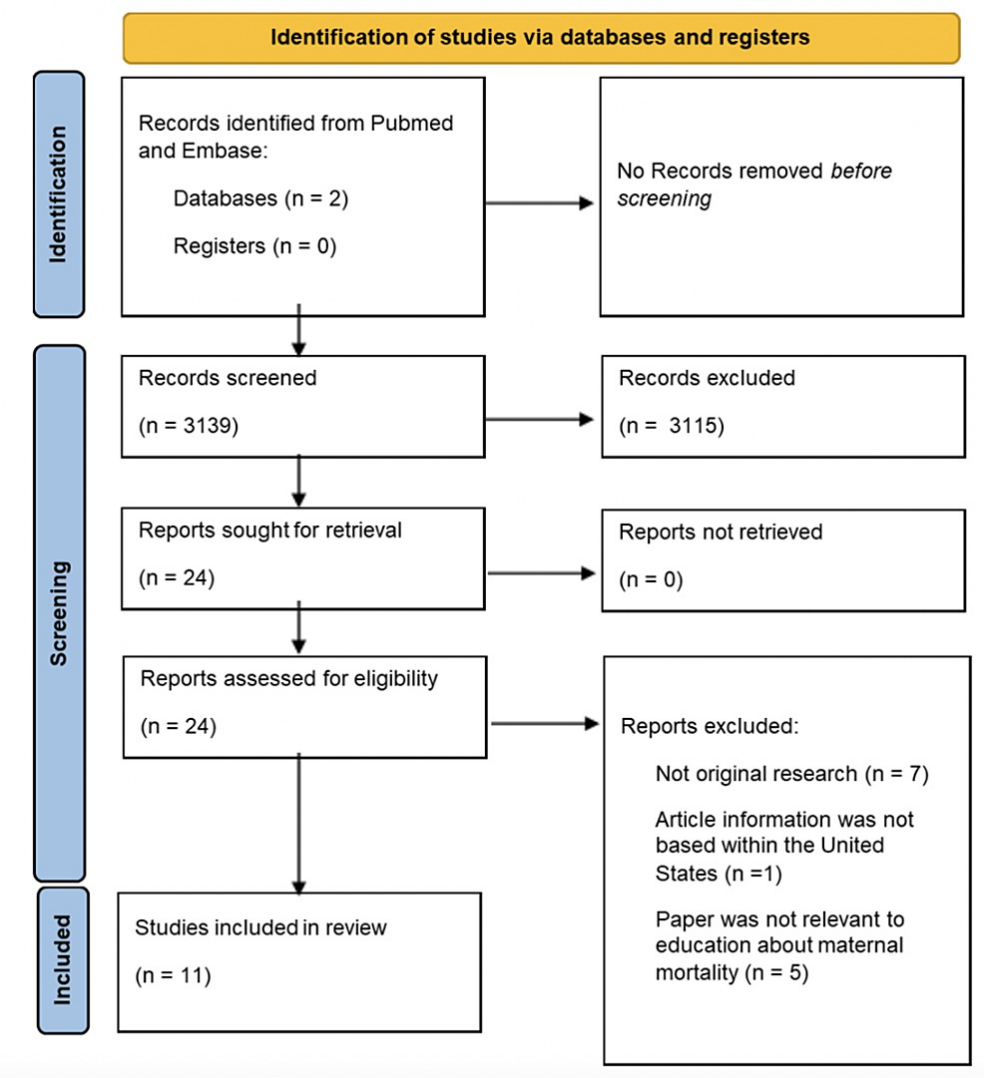
PRISMA flow diagram PRISMA, Preferred Reporting Items for Systematic Reviews and Meta-Analyses

Following this, all researchers completed a full-text review of each of the 24 articles. The final selection yielded 11 articles that were ultimately included after the full-text review. Of the articles that were determined to not meet the inclusion criteria after a full-text review, some were excluded for not being original research or for not being conducted in the United States [8-10] (Figure 1). Reviewers synthesized purposes and conclusions from all 11 articles (Table 2).

**Table 2:** Articles included after full-text review

Authors, Year	Title	Purpose	Conclusion	Database
Adams and Young, 2022 [11]	Perceptions of postpartum teaching and knowledge of warning signs among black mothers	Conduct a survey of Black women who gave birth in the last year	Only 54.4% of participants were said to have been counseled on warning signs. 25% of participants could name no warning signs	Embase
D'Oria et al., 2016 [12]	Strategies to reduce maternal mortality during the first year after birth	Discuss opportunities to reduce maternal mortality	Women of childbearing age should be asked at every visit if they have recently been pregnant	Embase
Hernandez et al., 2018 [13]	Pregnancy-related deaths, Florida, 1999–2012: opportunities to improve maternal outcomes	Analyze pregnancy-related deaths in Florida and advise on methods to reduce rates of maternal mortality	In more than 40% of pregnancy-related deaths, the primary factors were deficits in provided health care	PubMed
Logsdon et al., 2018 [14]	Do new mothers understand the risk factors for maternal mortality?	Determine understanding of maternal mortality in new mothers	There are areas for improvement in the education provided for new mothers	Embase
Martin et al., 2022 [15]	Maternal early warning criteria predict postpartum severe maternal morbidity and mortality after delivery hospitalization discharge: a case−control study	Analyze utilization of maternal mortality warning criteria in the postpartum period	Women who had early warning signs during the postpartum period were more likely to encounter maternal mortality	Embase
Morton et al., 2019 [16]	Translating Maternal mortality review into quality improvement opportunities in response to pregnancy-related deaths in California	Determine opportunities for improvement in causes of maternal mortality in California	Identified themes that were related to medical facility and ability to educate women about risk factors	PubMed
Suplee et al., 2017 [17]	Nurses’ knowledge and teaching of possible postpartum complications	Evaluate postpartum nurses’ awareness of maternal mortality and education given to women prior to discharge	67% of nurses educated women on warning signs for less than 10 minutes	Embase
Suplee et al., 2016 [18]	Discharge education on maternal morbidity and mortality provided by nurses to women in the postpartum period	Analyze postpartum nurses’ methods of providing education to women prior to discharge	There was not consistent education given to each woman	Embase
Suplee et al., 2017 [19]	Improving postpartum education about warning signs of maternal morbidity and mortality	Develop teaching for nurses to educate mothers on maternal mortality warning signs	Nurses stated that discharge education checklist was useful and they were satisfied with patient comprehension	Embase
Tucker et al., 2021 [20]	Comprehensively addressing postpartum maternal health: a content and image review of commercially available mobile health apps	Judge peripartum applications available on health education information provided and inclusivity	Only 45% of applications used evidence-based information and only 45% included peripartum health risks	PubMed
Vernon and Yang, 2022 [21]	Implementing a self-monitoring application during pregnancy and postpartum for rural and underserved women: a qualitative needs assessment study	Assess qualitative needs of underserved women in Georgia and determine views on proposed home health monitoring application	Feedback will allow for improvement in application and increase postpartum education and care of underserved mothers	Embase

Results

The 11 final articles demonstrated that no original research between January 2012 and September 2022 studied or made connections between maternal mortality and education on maternal warning signs in the state of Alabama.

Assessment of Maternal Knowledge

The current level of knowledge of maternal warning signs possessed by mothers demonstrates the gaps in the education provided. To elucidate this, a study from an academic medical center in Kentucky surveyed new mothers on their knowledge of maternal mortality risks and course of action [14]. Participants could identify warning signs such as heavy vaginal bleeding and severe swelling of the extremities and face, but less recognized shortness of breath, vaginal blood clots larger than a baby’s hand, and a fever as significant causes of maternal mortality. Only 42.5% of mothers would call 911 if they experienced one of these symptoms, and only 38.3% understood that complications from pregnancy could continue for up to a year following parturition. The overlooked information leaves a gap in which serious risks of maternal mortality may occur, as delays in seeking treatment have been directly associated with maternal deaths [16].

A study conducted in St. Joseph County, Indiana, examined the education black mothers received concerning maternal mortality warning signs. The results found that only 54.4% of participants were counseled on warning signs [11]. It was also determined that 25% of participants could not recognize any warning signs and that no participant could name more than five signs. These results again demonstrate a significant lack of proper education being provided to new mothers. The authors of the article emphasized that each patient needs to be thoroughly informed about the potential symptoms they could experience that would require immediate medical attention to prevent serious complications, including death. Furthermore, a retrospective case study of patients from 2013 to 2020 in Louisiana found that women who had higher instances of maternal mortality and morbidity also had increased early warning signs, such as significant deviations from normal vital signs [15]. This association emphasizes that education on warning signs could lead to receiving early care and reducing associated rates of maternal mortality.

Nurses as Educators

While some studies have demonstrated the lack of education given to mothers, others have attempted to find solutions through a hospital’s infrastructure. Multiple studies from Suplee et al. emphasized that registered nurses are present in the first moments following birth and as such are critical in informing patients about warning signs [17-19]. Despite finding that most nurses are providing patients with at least a partial education about postpartum complications, there are wide variations in the information they are sharing and how they are sharing it [18]. Of the nurses included in the study, 25% could not name the three leading causes of maternal mortality, and the leading cause of maternal mortality, cardiac disease, was not mentioned by any nurse. Knowing this, maternal education should be standardized and more informed by original research [19]. Suplee et al. demonstrated this concept by developing a 10-year initiative that provided postpartum mothers with standardized evidenced-based materials and teaching nurses what topics to focus on when providing discharge instructions. Nurses noted that the checklist and materials developed by the group assisted them with providing complete education to mothers and noticed that mothers had improved comprehension of the information being taught to them [19]. Furthermore, while examining nurses’ knowledge of maternal mortality, only 15% of nurses were aware of the most current rates of maternal death in the United States [17]. If health care providers and nurses are not up to date with the information themselves, their patients will bear the consequences of being uninformed.

In an additional effort to address the lack of information all mothers are given, an article sought to ensure that all patients at potential risk are identified. With an act as simple as asking every female patient of childbearing age if they are currently pregnant or have been pregnant within the last year, health care providers can drastically improve the number of women who receive maternal education [12]. By identifying which patients could be at risk of maternal mortality, providers can then share the necessary education on warning signs of serious complications. This association emphasizes that education on warning signs could lead to receiving early care and reduce associated rates of maternal mortality. Maternal mortality should not be solely addressed at health care visits related specifically to pregnancy.

Identifying Risks and Developing Recommendations

While examining causes of PRDs in Florida from 1999 to 2012, the Florida Pregnancy-Associated Mortality Review (PAMR) Committee developed specific recommendations to improve maternal outcomes. Principal contributing factors for PRDs included morbid obesity and late/no prenatal care. The five leading causes of death in this study were hypertensive disorders, hemorrhage, infection, cardiomyopathy, and thrombotic embolism. Of these five leading causes of PRDs, 42.5% of cases were identified to have one clinical or health care system quality care issue, which included coordination and communication. Based on these findings, PAMR recommendations for each leading cause of death included individual and community factors that focused on improving patient awareness of significant symptoms, educating high-risk patients on warning signs, and increasing community awareness of potentially harmful behaviors [13]. More comprehensive patient education can be easily implemented and result in tremendous impacts on rates of maternal mortality.

Providing Education to Enable Mothers’ Postpartum Health

Previous articles have demonstrated the gaps in maternal warning sign education, leading some researchers to determine ways to implement educational enhancements. For one method of improving patient education, an application to be used for remote pregnancy and postpartum monitoring has been developed [21]. Through daily blood pressure monitoring and weekly assessment of weight and mental health, the application advises when a result calls for seeking care. This allows patients to be actively involved in their health care and promptly address any potential warning signs. Additionally, health care facilities could incorporate mobile applications, already established by outside companies, into patient care. These applications provide information on maternal mortality risk factors as well as other helpful general maternal healthcare knowledge [20]. The ability of women to easily access information on their phones could improve their knowledge of potential warning signs.

Discussion

There is a demonstrated need for data supporting the efficacy of patient education in reducing rates of maternal death. Previous research has focused on categorizing the causes and trends of maternal mortality in the United States [8,22]. However, few have studied the effects of implementing recommendations, such as increased patient education, on rates of maternal mortality. Moreover, even fewer studies have attempted to capture statistical data that may better frame how patient education initiatives could lower the maternal mortality rates.

An urban hospital in Ghana designed an observational study to assess patients’ knowledge of preeclampsia, eclampsia, and associated warning signs from provider counseling given in the hospital. Patients who received complete counseling from providers demonstrated higher scores on knowledge assessments. In addition, a higher level of education was associated with higher assessment scores [23]. Within the United States, You et al. completed a randomized controlled trial assessing the effectiveness of a preeclampsia educational tool in comparison to a standard pamphlet addressing preeclampsia on patient knowledge of the disease. Patients who received the graphics-based educational tool scored significantly higher on the disease questionnaire in comparison to those who received the standardized pamphlet or no information at all. What may be most interesting from this trial is that improved understanding from this group was evident among women without adequate health literacy [24]. These trials demonstrate the power of patient education on maternal morbidities and the potential impact it could have on maternal mortality rates.

The Alabama Maternal Mortality Review Committee (AL-MMRC), launched in 2018, reviews statewide data and makes specific recommendations regarding how to decrease the rates of maternal mortality [2]. While analyzing maternal deaths between 2016 and 2017, they determined that more than 56.3% of deaths were preventable. Additionally, the AL-MMRC found that the main causes of death were cardiovascular events, substance overdose or toxicity, and infections. The AL-MMRC stated that providing education to patients and their families was a crucial way to decrease the risk of maternal mortality and emphasized that specific education on warning signs of potentially life-threatening illnesses was needed.

CDC developed the HEAR HER campaign to expand awareness of crucial warning signs related to maternal mortality [25]. The campaign website includes a quiz to test one’s knowledge of maternal mortality warning signs, printable resources that can be shared with patients, and materials for health care providers. Between this CDC national movement and state-specific programs, there is demonstrated awareness of the need to decrease the rates of maternal mortality. An examination of Florida efforts to reduce maternal mortality between 2001 and 2014 found that increasing specific public health program funding, including funds for educational material, by 10% would lead to a decline in maternal mortality by 3.9% [26]. It is imperative that health care providers become the bridge to connect their patients to these resources.

Matching health education to the mother's literacy level is key to improving patient comprehension of warning signs, especially as populations with low health literacy rates tend to also have increased rates of maternal mortality. In places such as Alabama, developing maternal education materials that are factual, accessible, and easy to understand is crucial in increasing health knowledge and preventing adverse events [12]. If these patients were counseled on these warning signs, they potentially would have sought out medical care sooner and had better health outcomes.

This scoping review has limitations. The decision to only use PubMed and Embase could have resulted in relevant articles being missed. Additionally, by choosing to focus on the United States, and specifically Alabama, pertinent data from other countries have been left out. Furthermore, while the initial blinded abstract review proved effective, it may have resulted in excluding articles due to lack of information in the abstract and reviewer bias.

## Conclusions

While the 11 final articles do not include original research conducted in Alabama, they do signify research being conducted within the United States. Maternal mortality rates continue to increase within the United States, and while multiple techniques have been discussed and attempted, most are unsuccessful. The United States must consider other options to ameliorate this public health crisis. This systematic scoping review attempts to discuss a compelling solution to such a disastrous statistic. Patient education may be instrumental in enabling women to not only understand their own health but also acknowledge teachable warning signs to the leading causes of maternal mortality. While there are multiple options for patient education -- for example, provider counseling, pamphlets, and videos -- some options may not be available to all patients and providers in certain areas across the United States. There are multiple areas in Alabama where expecting and postpartum mothers suffer from poverty and extraneous circumstances that make it difficult for them to manage their health. This puts great strain on the state and its population. However, this does not hinder the opportunity for providers to affect their patient’s health. If providers take the opportunity to assess their community by including women in the development of educational materials, it will ensure that the information is being conveyed appropriately and will enhance patient comprehension. By providing education to mothers through pamphlets or free classes given through volunteers, such as medical students and physicians, the major burden of maternal mortality can be decreased through cost-efficient methods. In conclusion, this review emphasizes the need for further research to directly assess the long-term impacts of patient education on maternal mortality outcomes not just in the United States but in one of the country’s highest areas of maternal mortality, Alabama.
